# Autism and anaesthesia: a simple framework for everyday practice

**DOI:** 10.1016/j.bjae.2024.01.002

**Published:** 2024-02-23

**Authors:** S. Brown, K. Rabenstein, M. Doherty

**Affiliations:** 1 Surrey and Sussex Healthcare Trust, Surrey, UK; 2 East Sussex Healthcare NHS Trust, Hastings, UK; 3 University College Dublin School of Medicine, Dublin, Ireland; 4 Our Lady's Hospital, Navan, Ireland

**Keywords:** anaesthesia, autism, reasonable accommodations

**Learning objectives**

By reading this article you should be able to:

• Explain the specific challenges faced by autistic patients presenting for anaesthesia.
• Recognise when patients of all ages may be autistic.
• Perform an autism-aware preoperative assessment.
• Optimise communication and provide reasonable adjustments for autistic patients.

**Key points**

• At least 1–2% of people are autistic. Most are adults without intellectual disability. Many are unrecognised.
• Autistic people are more likely to have co-occurring medical conditions; they experience well-described barriers to healthcare, adverse health outcomes and premature mortality.
• Illness or injury may present atypically and delayed presentation is common.
• Person-centred adaptations improve the experience of perioperative care.
• “Autistic SPACE” offers a framework for meeting the needs of autistic patients: Sensory needs, Predictability, Acceptance, Communication, Empathy.

In common with other published articles, in this review we use identity-first language (autistic adult, autistic child) rather than person-first language (person with autism). Although medical training generally dictates the use of person-first language, identity-first language is the overwhelming preference of autistic people.^1^

At least 1–2% of people are autistic.^2^ In anaesthesia, we tend to consider autism a paediatric condition, often associated with intellectual disability or the need for premedication.^3^ In reality, autistic people present for anaesthesia at all ages and stages of life. Despite increasing awareness and recognition, particularly in adults without learning disability, most autistic adults remain undiagnosed.^4^ Improved awareness has led to an increased prevalence of autism, particularly in women. Significant numbers of healthcare professionals are also identifying as autistic. All anaesthetists will encounter autistic patients (and colleagues) in their routine clinical practice.

Autism can be defined as a lifelong neurodevelopmental difference that influences the way a person interacts and communicates with others and experiences the world around them.^5^ These differences may manifest as unconventional social communication or social interaction, sensory sensitivities, focused interests and repetitive behaviour.^6^ Autistic features share common themes, but each individual will present with a personal profile of strengths and challenges which fluctuate according to circumstances.

When environmental and social demands exceed a patient's capacity to fit into a largely non-autistic world, such as when unwell and vulnerable in an unfamiliar environment, differences may become more evident and the need for accommodations increases.

Autistic people are more likely to experience co-occuring medical conditions and on average, die younger than non-autistic people.^7^^,^^8^ Well-described barriers to healthcare access may lead to adverse health outcomes.^9^ Previous negative experiences with healthcare act as an independent barrier to future healthcare for autistic people.^10^

Autistic people are legally protected by both the Equality Act (2010) and the Autism Act (2009). As medical professionals we have a legal obligation to provide reasonable adjustments to accommodate individual needs.^11^^,^^12^ A framework to guide understanding, care and accommodations for autistic people using the acronym ‘SPACE’ has been proposed for use in healthcare settings.^13^ It is adapted here for use in anaesthesia (Fig. 1).^13^

**Fig 1:**
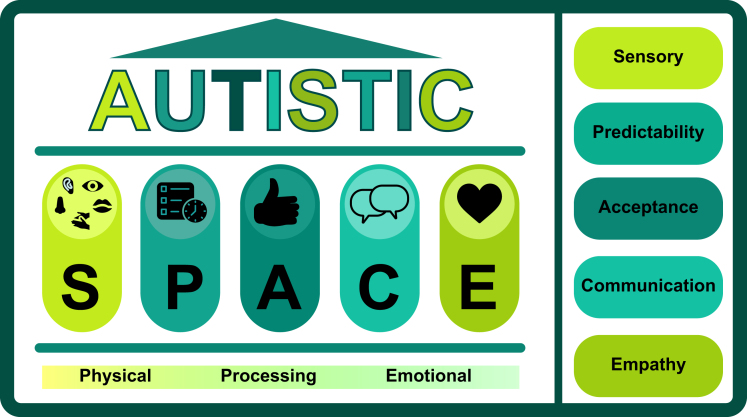
Autistic SPACE framework.

In this review we aim to give an overview of autism and perioperative considerations, including communication strategies and suggestions for addressing sensory needs and preferences for predictability. Hospital environments may be daunting for many patients, but this is magnified for autistic people because of the lack of predictability, loss of routine and sensory challenges. Simple adjustments can vastly improve the experience, which may have an impact on outcomes.^9^

## Prevalence, identification, diagnosis and disclosure of autism

The current prevalence of autism in adults in England is estimated to be 1.1% although this may be a significant underestimate.^2^^,^^14^ More recent data show that 2.7% of American school children are autistic with corresponding prevalence in Northern Ireland documented as 4.7%.^15^^,^^16^ Changing prevalence is more likely a result of increasing recognition and case-finding methodology, rather than a true increase.^17^ Recognition of non-stereotypical presentations, particularly autistic women and non-binary people, accounts for the reduction of the historical male preponderance, potentially towards parity.^18^ Although autism is neither a mental disorder nor an intellectual disability, there is an increased incidence of intellectual disability amongst autistic patients, with current prevalence estimated at 15–30%.^4^^,^^19^

Autism can be either syndromic or non-syndromic. Specific genetic syndromes such as fragile X, Angelman syndrome, Phelan McDermid syndrome, neurofibromatosis type 1 or tuberous sclerosis complex are associated with autism.^20^ Down syndrome is also associated with an increased prevalence of autism.^21^ Autism-specific accommodations must be considered for such patients. Epilepsy is a common coexisting condition, with a pooled prevalence of 10%. Rates are higher in autistic adults than autistic children and epilepsy is particularly prevalent among those with syndromic forms of autism and coexisting intellectual disability.^22^

Relying on a formal diagnosis of autism may not be a robust strategy to identify all autistic patients. The current autism diagnostic criteria focus exclusively on the deficits associated with autism leaving many autistic people unrecognised by themselves or professionals because of diagnostic phrasing and stereotyping.^6^^,^^23^ Patients may also fear stigma and stereotypes associated with the diagnosis. Many who self-identify as autistic may choose to avoid seeking a diagnostic assessment. Others cannot access a diagnosis because of service constraints. Those diagnosed may not feel comfortable to disclose. Instead, many attempt to ‘mask’ or camouflage autistic traits to varying degrees. Masking is more common in autistic women and may lead to anxiety, depression and suicidality. Patients may be experiencing intense internal distress, while maintaining an impassive demeanour.

Clinicians are better at noticing ‘difference’ either medically or in social interactions, than determining if someone fits diagnostic criteria. Anaesthetists often recognise patients who appear unconventional or ‘different’ but may not realise that the difference may signify the patient is likely to be autistic. The principles discussed here may be applicable in such cases. For a detailed account of recognising autistic patients in healthcare settings see reviews by Doherty and colleagues.^24^^,^^25^

## Coexisting health conditions, adverse outcomes and barriers to healthcare

Autism is associated with a mortality gap of 16–30 yrs.^8^ Difficulties accessing primary care may lead to delayed presentations to emergency care. Autistic people are three times more likely to require hospital admission after emergency department presentation and twice as likely to die as inpatients after emergency admission.^26^ Mortality is increased across all diagnostic categories.^8^

When performing a preoperative assessment, the anaesthetist should be aware of the co-occurring conditions that may be present. Obesity, diabetes and hypertension are more common, increasing the rate of secondary complications.^27^ Immune-related conditions, gastrointestinal and sleep disorders, dyslipidaemia and Parkinson's disease are also more prevalent. Mental health conditions are significantly more frequent, particularly depression and anxiety.^28^ Suicide is common, as is self-harm.^8^^,^^29^ Other neurodevelopmental conditions commonly co-occur, in particular attention deficit hyperactivity disorder (ADHD) which occurs in a significant proportion of autistic adults and children.^30^ There is an association with joint hypermobility syndromes such as Ehlers–Danlos, autonomic dysfunction (including orthostatic intolerance) and increased rates of chronic pain.^31^^,^^32^

Many of the well-described healthcare access barriers experienced by autistic people overlap with the patients' perioperative pathway. Doherty and colleagues reported that 80% of autistic respondents experienced difficulty visiting a general practitioner when required, and found correlations with self-reported adverse health outcomes.^9^

Autistic people tend to avoid using the telephone, which is one of the most common barriers as use of the telephone is ubiquitous in healthcare.^9^^,^^33^ Other difficulties arise from the sensory environment and lack of understanding by medical and ancillary staff. The consequences of such access barriers are not trivial, with late presentations and inability to follow up on specialist referrals. Over a third of autistic people reported inability to access care for potentially serious or life-threatening conditions.^9^

Delivery of high-quality anaesthesia care for autistic patients requires autism-specific accommodations delineated in the Autistic SPACE framework below. Using such a framework may lessen the impact of healthcare-related trauma. For example, a difficult induction may be a traumatic memory for a non-autistic person, but it may prevent an autistic person seeking care for future life-threatening conditions.

## Autistic SPACE as a framework for understanding and accommodations

The autistic spectrum is not linear. Support needs vary considerably between autistic people and in different environments; relatively low at home when relaxed, but can significantly increase when unwell in hospital. Although autistic profiles are variable, there are common aspects that can be addressed, and attention to such aspects will greatly improve the perioperative experience.

### Sensory needs

Sensory sensitivities are paramount for autistic people and can render a healthcare environment totally inaccessible.^34^ Noise, lights, smells and tastes are perceived differently compared with non-autistic people. Touch is also perceived and tolerated differently, which has implications for clinical examination. Unexpected touch is particularly unwelcome, but firm touch is usually better tolerated than light touch. Sensory challenges also impede capacity to communicate.^9^ See Table 1 for details of sensory differences and Table 2 for suggested accommodations.

**Table 1:** Autistic sensory differences, adapted from Autistic SPACE.^13^

Sensation	
Sight	Visual sensitivities are common. Bright lighting (particularly fluorescent) is a common challenge. Visual stimuli that may go unnoticed by non-autistic people, such as the flickering of fluorescent lighting or screens, may cause sensory stress. Operating theatres often have bright ceiling lighting, operating lights and many flashing lights and warning alarms associated with monitoring equipment.
	
Sound	Auditory sensitivities and auditory processing differences are common. Environmental noise, particularly when sudden or unexpected, can cause intense distress. Theatres are inherently noisy environments, with anaesthetic machine and monitoring alarms, clashing of metal surgical trays during preparation and background chatter. Environmental noise can make conversations difficult to process and will exacerbate autistic overwhelm.
	
Smell	Autistic people are often highly sensitive to smell and may perceive odours that others do not. Common and usually inoffensive smells may be perceived as highly noxious. Diathermy cauterisation, surgical prep, chlorhexidine cleaning sticks may cause difficulties for autistic staff and patients.
	
Touch	Tactile sensitivities range from inability to tolerate the sensation of certain fabrics including theatre gowns, sheets and blankets to an inability to be touched, particularly by strangers. Monitoring attached on arrival is often cold (BP cuff/ECG electrodes); interventions we perform are uncomfortable or painful.
	
Temperature	Sensitivity to temperature is common. Theatres are often cold so patients may benefit from additional blankets, but the range of easily tolerated temperatures is likely to be person-specific. Many prefer a cold environment and will reject interventions such as forced air warmers.
	
Interoception and pain	Some autistic people have difficulty interpreting internal bodily sensations. This can lead to difficulties noticing hunger, thirst, tiredness, or a need to urinate or defecate.
	Patients can sometimes have reduced pain sensitivity, but more commonly they have increased pain sensitivity coupled with difficulty communicating their pain.

**Table 2:** Recommendations for supporting Autistic SPACE in practice. Adapted from Autistic SPACE.^13^

SPACE framework aspect	Recommendations for implementation
**Sensory**	
Sight	• Reduce anaesthetic room lighting
	• Induce in anaesthetic room rather than theatre
Sound	• Reduce background noise (preparing surgical trays in prep room rather than theatre)
	• Avoid multiple people talking to the patient at once
	• Allow/suggest the use of noise-cancelling headphones and ear plugs
Smell	• Avoid strong smells if possible—cleaning products/air fresheners etc.
Taste	• Respect sensory preferences (e.g. liquid medication)
	• Consider preference for inhalation or i.v. induction
Touch	• Ask about tactile preferences and modify examination technique
	• Avoid casual touch
	• Consider allowing own clothes to theatre with gown over the top
	• Sensory aids such as weighted blankets
Temperature	• Consider environmental temperature and individual thermal preference
	• Offer or avoid additional blankets or forced air warmers as preferred
Interoception and pain	• Ask directly about internal sensations but understand that answering may be difficult
	• Acknowledge and address any complaints of pain, even if they do not appear in pain
	• Adapted pain scales may be required
**Predictability**	• Give written information in advance—accurate signs/maps etc.
	• Explain carefully what to expect and be as accurate as possible with time frames
	• Allow opportunity for a tour before surgery
	• Allow waiting in a familiar environment (e.g. outside/in own car) if possible
	• Ensure care is provided by staff familiar to the patient where possible
**Acceptance**	• Neurodiversity-affirmative approach beneficial
	• Understand autistic stimming and monotropic thinking patterns
	• Facilitate need for detailed, factual information
	• Understand distress behaviour
**Communication**	• Understand autistic verbal and non-verbal communication differences
	• Know that communication ability is reduced by anxiety and sensory stress
	• Use clear unambiguous communication
	• Promote use of augmentative and alternative communication (AAC)
**Empathy**	• Recognise that autistic people feel empathy but may display it differently
	• Empathy towards autistic patients may be more challenging for non-autistic healthcare providers
**Physicalspace**	• Allow for increased personal space
	• Avoid proximity to other people where possible
**Temporal space**	• Allow increased time to respond to questions
	• Allow increased time for decision-making
**Emotional space**	• Expect differences in emotional expression
	• Allow space to recover, without additional input, if distressed

### Predictability

Much of the anxiety experienced by autistic people relates to intolerance of uncertainty and this accounts for the preference for familiarity and routine. New environments and unknown people are challenging. Waiting is difficult, particularly when it is for an unknown duration.^35^

### Acceptance

As the autistic experience of the world differs from that of non-autistic people, so too does autistic behaviour. Understanding what is usual for an autistic patient facilitates care. Repetitive behaviour, which is common to autistic people, is termed ‘stimming’. It varies in manifestation and function, but may include hand flapping, whole body movements or the subtle use of fidget tools. In stressful situations, anxiety may be alleviated by stimming, so we advise encouraging the use of whatever tools a patient finds beneficial.

A ‘monotropic’ cognitive style is a common autistic characteristic, and relates to the autistic tendency to have deep interest in single topic areas. It can be helpful to understand and accommodate this tendency during the perioperative period. A hyperfocused interest in particular (often niche) subjects may be used to develop rapport, or used as distraction where required. Monotropism can also manifest as perseveration or repeated questioning, and in such cases we advise responding with patient, repeated answers. This becomes easier once caregivers understand the underlying nature of the interaction.^36^

Accepting, understanding and accommodating the needs of an autistic patient will reduce distress, maximise cooperation and minimise the risk of what is often termed ‘challenging behaviour’ or autistic meltdown, leading to a smoother experience of perioperative care for the patient, family and staff.

### Communication

Autistic people communicate differently to non-autistic people, regardless of age or stage of development. Many converse fluently when relaxed but may struggle to form words or articulate thoughts under pressure. Some may speak only a few words and others do not develop speech at all. Many non-speaking or minimally speaking autistic people use augmentative and alternative communication (ACC) which may be unfamiliar to care providers.^37^ Healthcare professionals should ascertain and facilitate a patient's usual means of communication, and the use of AAC should be offered when patients who usually communicate verbally find themselves unable to do so. A flexible approach to communication and the use of written text or phone apps such as ‘Emergency Chat’ can be helpful.

Those who usually speak but are unable to do so when under stress may need to write, type or text instead, but this does not imply lack of capacity. If you need to formally assess a patient's capacity, it is best to conduct this assessment with the presumption of competence, but if unable to consent, local guidelines should be followed. Where possible, facilitate assisted decision-making, with the help of a suitable supporter where appropriate.

Autistic people may use language that is idiosyncratic and tend to interpret spoken words very literally. Questions may be answered factually, omitting information that is not directly elicited. Clear unambiguous language is therefore necessary.

Non-verbal communication is also different. Eye contact, the use of gaze, gestures and posture can all lead to miscommunication if autistic ways of communicating are not appreciated. This is particularly relevant to clinical examination where the usual non-verbal signs of pain may not be evident. Healthcare professionals tend to judge the severity of a condition by familiarity with largely non-autistic presentations, leading to underestimation of illness in autistic patients.

### Empathy

It is a common misconception that autistic people do not feel empathy. Although empathy may be experienced or expressed differently, this myth has led to the assumption that autistic people do not feel empathy at all. The reality is that many autistic people describe a state of ‘hyperempathy’ which can be overwhelming and leads to an involuntary ‘shutdown’ to manage the intensity of emotion. Mutual misunderstanding occurs between autistic and non-autistic people, which has been termed the ‘double empathy problem’.^38^ It requires specific effort from non-autistic healthcare providers to empathise with those whose experience of the world and means of interacting is significantly different to their own. It is easier for autistic healthcare providers to understand, communicate and develop rapport with other autistic people, whether patients or colleagues.^39^

The concept of Autistic SPACE can usefully be applied to three other domains of autistic experience, using the mnemonic now familiar to all anaesthetists: ‘PPE’.

### Physical ‘SPACE’

Autistic people commonly require more personal space than non-autistic people and find close proximity to other people distressing. Tactile defensiveness is common; an autistic person may react negatively when touched or crowded, particularly when stressed.

### Processing ‘SPACE’

The time taken to process new information is generally longer than for non-autistic people. Unexpected changes, choices or decisions may require longer to process or respond to. Healthcare providers might prematurely assume a lack of understanding or capacity. Understanding the differences in autistic processing and having patience to wait may result in a more appropriate outcome.

### Emotional ‘SPACE’

Autistic people express and process emotions in unconventional ways. Approaches considered helpful with non-autistic patients, such as touch or general reassurance can be perceived differently by autistic people and inadvertently escalate distress. The best approach with a distressed autistic person is to minimise sensory and social input by avoiding unnecessary conversation or touch.

## Applying the Autistic SPACE framework to the perioperative pathway

Here we focus on specific areas of anaesthetic care which may differ for autistic patients or where the importance of aspects of standard care may be different.

### Advanced planning

Improving the experience for autistic patients starts in the preoperative planning phases. Advanced planning should involve multidisciplinary teams with appropriate dissemination of individual patient plans to all relevant team members. Autistic patients may have an autism health passport, which might provide useful information on communication needs and preferences, how pain and distress are experienced, individual sensory needs and how best to offer help in various circumstances. Methods of familiarising a patient with what to expect from their hospital stay range from the RCoA's patients' information leaflets (see Box 1 for examples) to online videos or giving a tour of the anaesthetic room, operating theatre and recovery room. The aim is to maximise predictability, reduce uncertainty and thereby allay anxiety.

Box 1 **Suggestions for details to be included in an information leaflet for autistic patients.**

• Contact details for the department via different means (e-mail, text, phone or in-person)
• Online/text-based options to book and alter appointments
• Preoperative assessment clinic details including virtual options
• Written detailed preoperative clinic instructions and what to bring to appointments
• Clear directions, maps and parking information
• Photographs or a link to a video tour of the hospital/department

Alt-text: Box 1

Where possible, the patient should be scheduled early on an operating list and ideally as a day case if the procedure is suitable. This approach will minimise the time spent in an unfamiliar environment and facilitates earlier discharge. Some autistic adults who live socially isolated lives may not have a readily identifiable appropriate person to act as an escort home after the surgical procedure, and special efforts may therefore need to be made to create a suitable, individualised discharge pathway for them.^9^ When scheduling postoperative follow-up, discuss the preferred method of contact for the appointment and offer suitable options such as online contact. If telephone is unavoidable, autistic patients may prefer to call the hospital rather than receive a call.

## Preoperative assessment

Autistic patients are often well informed regarding their health and will provide required information when requested, but may not offer additional details unless specifically asked. Begin by determining communication capacity, needs and preferences and whether a carer or advocate is needed. It is preferable to take a history from the patient, but collateral history is beneficial. It is best to take a routine anaesthetic history including coexisting conditions and medications in conditions of privacy. It is essential to ascertain what an autistic person's anxiety response is and what their distressed behaviour may look like.

Understanding a patient's triggers and responses will minimise distress. Attention to the sensory environment and an opportunity for the patient to familiarise themselves with the location and personnel will maximise co-operation. The use of restraint should always be as a last resort and adequate preparation should avoid any need for this.

Therapeutic holding occurs not uncommonly at induction of anaesthesia in paediatric practice when children are uncooperative. Unfortunately, autistic children are up to three times more likely than non-autistic children to experience restraint in the emergency department.^40^ This increased use of restraint is also likely to be the case in the operating theatre, particularly for autistic children with coexisting intellectual disability. The use of restraint is deeply traumatic for patients, parents and staff; practitioners should always strive to avoid it, but the potential need for restraint to achieve a safe induction of anaesthesia requires consideration and potentially discussion at the preoperative assessment. The use of restraint with autistic children is discussed in detail elsewhere.^3^

Very occasionally, therapeutic holding with consent may be applicable for selected autistic adult patients. This may occur with autistic adults who have capacity, want to consent to surgery but are unable to cooperate in the moment. Repeatedly abandoning a needed procedure may be more traumatic than offering the option of assistance to stay still by gentle restraint, provided the patient understands the proposed course of action and gives truly informed consent.

Consider carefully in advance any scenario where non-consensual restraint may become necessary. Safety for the patient and for staff is key in such situations so advance preparation is essential (see induction, below).

### Choice of anaesthetic technique

Clearly explain the options for general anaesthesia including the choice between i.v. and inhalational induction where appropriate. Whereas regional anaesthesia may be highly beneficial for postoperative pain relief, the experience of paraesthesia and loss of sensation or function may be distressing, in which case multi-modal analgesia including local infiltration may be preferable. The benefits of pain management devices such as patient controlled analgesia pumps may be outweighed by intolerably frequent and noisy alarms. Whichever technique is chosen, explain the procedure clearly and avoid false reassurances that an intervention will be painless, as an autistic person may take this literally, leading to loss of trust and potentially distress reactions.

### Premedication

Although sedative premedication is less commonly used in modern anaesthesia, particularly for adults, autistic patients of any age, particularly those with previous traumatic experiences of healthcare may benefit from premedication in the ward before transfer to the operating theatre.

The choice of premedication will be based on anaesthetist preference and any previous idiosyncratic or paradoxical reaction the patient may be known to have had.

Midazolam is most commonly used (oral/buccal) but it can be poorly tolerated because of taste. Clonidine (oral) is tasteless, and ketamine has a minimal taste. Dexmedetomidine can be administered by nasal atomiser if a patient is not amenable to oral administration.

Typical doses are^41^:

• Midazolam (buccal) 0.3 mg kg^−1^ (maximum 10 mg); onset time of 20 min, duration 30–45 min
• Dexmedetomidine 2 μg kg^−1^ (maximum 200 μg); onset time 25 min, duration 40–135 min
• Clonidine 4 μg kg^−1^ (max 200 μg) onset time 45–60 min, duration 45–90 min
• Ketamine 5 mg kg^−1^; onset time 10–15 min, duration 3 h

Make decisions about premedication, including the drug, dose and location it is to be given on an individual basis. Some patients, particularly those with a history of healthcare trauma or those scheduled for emergency surgery without the required time for familiarisation, may need sedation before transfer to theatre. Carefully consider the level of supervision required after premedication.

Offer topical local anaesthetic cream or ethyl chloride spray to all autistic patients regardless of age but be aware that sensory sensitivities may impact whether it is tolerated.

## Discussing the postoperative period

To reduce anxiety associated with uncertainty, provide the patient (and carer) with an outline of what to expect after surgery: where they will wake up; who will be present; and the likelihood of pain and other symptoms. Pain management requires discussion before surgery, including which pain scales would be most appropriate or if an alternative means of assessing pain will be needed.^42^ Postoperative recovery staff may need to use alternative assessment methods such as FLACC (faces, legs, activity, cry, consolability), FACES pain pictures or other non-verbal pain scales.

Although this review focuses predominately on advice for anaesthetists, it is important to consider the wider operating theatre team. The team briefing is an ideal opportunity to discuss list order amendments (to allow for peak effect of premedication) and potential difficulties that may arise at induction or emergence. Close communication with the ward/admission area is essential to facilitate timings of administration and arrival.

## Preoperative assessment area and the anaesthetic room

Before surgery, patients should wait in a side room, behind curtains or even off the ward to avoid a crowded, often noisy, unfamiliar environment. Be upfront if the duration before going to theatre is unknown. Avoid giving inaccurate estimates as any discrepancy between expectation and reality may cause confusion and distress. Where possible, minimise the time spent in the hospital environment, by staggering admission times or listing the patient first on the list.

Encourage the use of comfort items and sensory aids such as noise-cancelling headphones, including right up to induction of anaesthesia where feasible. It is helpful to have a supply of inexpensive sensory aids available; these can include disposable ear plugs, sunglasses and a selection of fidget tools or tactile sensory aids.

You may need to adopt a non-standard approach to relating to the patient. Healthcare providers commonly chat to patients to establish rapport; but for an autistic individual this may increase stress and overload and hence silence may be preferred. We advise establishing an individual's preference by asking directly.

### Induction of anaesthesia

Simple adjustments can significantly improve the patient's experience, the most important of which is remaining calm and confident, to minimise patient anxiety and distress. Induction should occur in a quiet space with the minimum number of necessary personnel present. Where possible, dim the lights and reduce the volume of monitoring alarms. If required, a supporter should accompany the patient, regardless of age. To ensure no interruptions while the patient is in the anaesthetic room, close the door and use signage such as ‘no entry’ according to locally approved practice.

Limit verbal interaction with the patient to a single individual where possible, in order to reduce the patient being overwhelmed. Keep conversation factual, unambiguous and reassuring rather than questioning or chatty. Encourage patients to talk about particular interests as a means of distraction, but silence is common when the autistic person is anxious, and forced conversation increases anxiety. If a patient has previously declined the option of silence but later appears anxious, it may be worth reconsidering silence. Expect autistic features and body language, such as lack of eye contact, stimming and vocal noises.

A hands-off approach can be helpful, such as letting the patient attach monitoring equipment and holding the oxygen mask themselves. If a patient has difficulty tolerating a mask, then wafting oxygen near but not touching the face may suffice if feasible. Performing multiple talks simultaneously may feel overwhelming to autistic patients, particularly when people approach from both sides at once, therefore we advise a sequential approach to preinduction tasks. Apply recommended monitoring as tolerated, but exercise clinical judgement. Explain noises, such as the tone of pulse oximetry = and mute alarms as appropriate. It is essential to attempt to identify a likely cause for any distress, either by asking directly or by visual clues.

Induction may require non-standard positioning (including in exceptional circumstances commencing induction with a standing patient). Safety is crucial in such cases and, as when anticipating non-consensual restraint, ensure that sufficient personnel are immediately available.

In some instances, autistic patients with coexisting severe or profound learning difficulties will require non-traditional sites for induction of anaesthesia or the use of premedication. This includes locations such as in the hospital carpark or in very rare cases in their home environment with an awaiting ambulance team, although this presents significant logistical and safety risks.

### Intraoperative management

Airway management should take account of dysmorphism associated with syndromic forms of autism. Autonomic dysfunction may occur, particularly with patients with joint hypermobility syndromes, therefore pay close attention to positioning to avoid joint dislocations. Guidance on anaesthesia for patients with hypermobility syndromes, including pain management challenges is detailed elsewhere.^32^^,^^43^

### Emergence from anaesthesia

Emergence is often the most challenging aspect of anaesthesia for autistic patients. As with all patients, adequate analgesia, antiemetics and rehydration are essential as these issues can often cause significant distress in the postoperative period and may impair the patient's ability to communicate.

Before emergence, reliably secure i.v. access (bandaged or splinted) and remove non-essential monitoring. Transfer the patient to a trolley asleep and consider the use of padded cot sides. Use clinical judgement regarding reconnecting any monitoring which becomes disconnected during emergence; similarly for displaced covers, albeit with attention to temperature.

Abrupt wakening with potential thrashing may occur. It is important to protect all involved from injury while minimising touch or restraint. All non-essential personnel should leave the area, and any noisy activity which routinely takes place at this time should pause.

Emergence should occur in a quiet environment, ideally with only one familiar voice using a single phrase such as ‘it's over, you're safe’, repeated slowly. Oxygen saturations allowing, wafting oxygen over the patient, rather than forcibly applying a mask to their face, is preferable.

## Postoperative recovery, discharge home and aftercare

Allow recovery from anaesthesia to occur in a dim, quiet environment, away from other patients, with alarm volume settings reduced or muted as appropriate. Patients should have a familiar person present once airway reflexes have returned, and be discharged back to the ward as soon as it is safe to do so. As autistic patients may not show typical signs of pain, it is important to enquire frequently about pain and provide analgesia as requested.

Relaxation of postoperative discharge criteria (such as the requirement to urinate after non-urological surgery) may be appropriate as the benefit of secondary recovery in the familiar environment of home may outweigh risk. Clear and unambiguous safety netting requires specific written advice as to when to seek assistance with wounds, pain, nausea and any other potential complications. Confirm communication preferences and appointment type for any follow-up before discharge.

Required reasonable accommodations are easier to implement on routine operating lists compared with emergency cases, but they are even more important when autistic people require emergency surgery. Priority scheduling as case-mix allows, highlighting that a patient is autistic at booking and at team brief and explaining clearly to the patient the unfortunate unpredictable nature of emergency lists will be helpful.

## Other clinical settings

Autistic patients may be encountered in other areas of anaesthetic practice, such as sedation, analgesia or anaesthesia for dental surgery, obstetric care or diagnostic procedures. Although the anaesthetic care of the autistic parturient or the critically ill autistic patient is beyond the scope of this paper, the principles outlined here should be applied.

## Autistic healthcare professionals

Autistic colleagues are numerous and to date mainly undiagnosed or undisclosed. Medicine and in particular, anaesthesia, self-selects for autistic strengths. Autistic professionals may also need reasonable accommodations, such as avoiding music in theatre and minimising unexpected changes to theatre lists or staffing rotas. Lived experience is valuable in understanding both professional and patient aspects of autism, and when shared with neurotypical peers can improve quality of care and team coherence. Including autistic team members in the care of autistic patients where feasible is sensible and can offer teaching opportunities.^44^

Despite insider knowledge of pathophysiology and healthcare systems, autistic healthcare staff face similar barriers accessing healthcare, demonstrating the urgency of improving care pathways with understanding and accommodations.^9^^,^^45^

## Conclusions

Autism is an under-recognised lifelong neurodevelopmental condition. The awareness of autism is greatest in paediatric practice, despite higher numbers within adults. Many autistic patients will have a diagnosis, although they may or may not disclose; many others will be unrecognised. Any patient presenting with an unconventional communication style or demeanour, monotropic interests, obvious anxiety, subtle repetitive movements or fidgeting may be autistic and is likely to benefit from the approach suggested.

Autistic people experience healthcare inequalities and a clear mortality gap. Healthcare providers should attempt to understand the perspectives of autistic people, including individual sensory sensitivities, communication requirements and decompensation style, in order to implement reasonable adjustments. Adapting communication, creating a predictable and tolerable environment and avoiding negative judgements may improve clinical outcomes.

In this review we aim to raise awareness of specific needs and challenges and offer potential solutions to optimise the perioperative pathway and minimise healthcare-associated trauma. As with planning for the patient with a difficult airway, the procedure is usually smooth with adequate preparation. Practical suggestions include using the Autistic SPACE framework, raising staff awareness with regular training (best led by a departmental autism champion) and engagement with local equality and diversity teams to create best practice guidelines.

By increasing understanding and providing accommodations we can improve patients' experiences and outcomes, while creating a better work environment for our neurodiverse teams.

## Declaration of interests

The authors declare that they have no conflicts of interest.

## MCQs

The associated MCQs (to support CME/CPD activity) will be accessible at www.bjaed.org/cme/home by subscribers to *BJA Education*.
